# Automated Cellular-Level Dual Global Fusion of Whole-Slide Imaging for Lung Adenocarcinoma Prognosis

**DOI:** 10.3390/cancers15194824

**Published:** 2023-10-01

**Authors:** Songhui Diao, Pingjun Chen, Eman Showkatian, Rukhmini Bandyopadhyay, Frank R. Rojas, Bo Zhu, Lingzhi Hong, Muhammad Aminu, Maliazurina B. Saad, Morteza Salehjahromi, Amgad Muneer, Sheeba J. Sujit, Carmen Behrens, Don L. Gibbons, John V. Heymach, Neda Kalhor, Ignacio I. Wistuba, Luisa M. Solis Soto, Jianjun Zhang, Wenjian Qin, Jia Wu

**Affiliations:** 1 Shenzhen Institute of Advanced Technology, Chinese Academy of Sciences, Shenzhen 518055, China; 2 Shenzhen College of Advanced Technology, University of Chinese Academy of Sciences, Shenzhen 518055, China; 3 Department of Imaging Physics, Division of Diagnostic Imaging, The University of Texas MD Anderson Cancer Center, Houston, TX 77030, USA; 4 Department of Translational Molecular Pathology, The University of Texas MD Anderson Cancer Center, Houston, TX 77030, USA; 5 Department of Thoracic/Head and Neck Medical Oncology, The University of Texas MD Anderson Cancer Center, Houston, TX 77030, USA; 6 Department of Pathology, The University of Texas MD Anderson Cancer Center, Houston, TX 77030, USA; 7 Department of Genomic Medicine, The University of Texas MD Anderson Cancer Center, Houston, TX 77030, USA

**Keywords:** embedded features, global fusion, cellular architecture, whole-slide image, survival prediction, lung adenocarcinoma

## Abstract

**Simple Summary:**

Lung cancer is the leading cause of cancer death in the United States and worldwide. Currently, deep learning–based methods show significant advances and potential in pathology and can guide lung cancer diagnosis and prognosis prediction. In this study, we present a fully automated cellular-level survival prediction pipeline that uses histopathologic images of lung adenocarcinoma to predict survival risk based on dual global feature fusion. The results show meaningful, convincing, and comprehensible survival prediction ability and manifest the potential of our proposed pipeline for application to other malignancies.

**Abstract:**

Histopathologic whole-slide images (WSI) are generally considered the gold standard for cancer diagnosis and prognosis. Survival prediction based on WSI has recently attracted substantial attention. Nevertheless, it remains a central challenge owing to the inherent difficulties of predicting patient prognosis and effectively extracting informative survival-specific representations from WSI with highly compounded gigapixels. In this study, we present a fully automated cellular-level dual global fusion pipeline for survival prediction. Specifically, the proposed method first describes the composition of different cell populations on WSI. Then, it generates dimension-reduced WSI-embedded maps, allowing for efficient investigation of the tumor microenvironment. In addition, we introduce a novel dual global fusion network to incorporate global and inter-patch features of cell distribution, which enables the sufficient fusion of different types and locations of cells. We further validate the proposed pipeline using The Cancer Genome Atlas lung adenocarcinoma dataset. Our model achieves a C-index of 0.675 (±0.05) in the five-fold cross-validation setting and surpasses comparable methods. Further, we extensively analyze embedded map features and survival probabilities. These experimental results manifest the potential of our proposed pipeline for applications using WSI in lung adenocarcinoma and other malignancies.

## 1. Introduction

Cancer mortality and morbidity remain fatal global burdens, among which lung cancer, the leading cause of cancer death in the United States and worldwide, has a higher risk of death than other cancers [1]. Lung adenocarcinoma (LUAD) is a common histological subtype of lung cancer and accounts for about 40% of all lung cancers [2]. However, LUAD is often diagnosed at an advanced stage. Despite significant efforts to improve LUAD treatment, survival rates are still suboptimal [3,4]. Precise survival prediction contributes to the mitigation of overtreatment and a reduction in economic costs [5].

Pathological assessments have been the gold standard for cancer diagnosis, and their role also extends to prognostication [6,7]. With the application of the whole-slide imaging scanner, digital pathologic images from tissue glass slides have markedly aided the development of computational pathology, including the prediction of the survival risk of patients [8]. This facilitates the study of more precise interconnections between pathologic image structures and the LUAD prognosis.

With the development of computing technology, numerous artificial intelligence studies have sought to automate tissue-level diagnosis [9,10,11], with some reports of deep learning matching pathologist-level performance [12,13]. Compared to cancer diagnosis, predicting treatment response or survival has thus far received less attention. Essentially, a cancer diagnosis is a classical classification task for a given glass or digital slide based on the examination of its morphology [14]. In contrast, patient prognosis is a more complex predictive problem that can be driven by both intrinsic (e.g., host anti-tumor immunity and performance status) and extrinsic (e.g., treatment and lifestyle) factors [15]. Pathological images provide a method for quantifying the intrinsic properties of the tumor and its host. However, the large scale of pathological slides and intratumor heterogeneity pose a significant challenge in distilling whole-slide image (WSI) patterns to predict survival. Existing WSI feature extraction methods can be categorized into two main paradigms: the patch-based approach [16] and the nuclei-based approach [17]. The patch-based approach first divides the WSI into several regular-sized patches, then utilizes a pre-trained convolutional neural network (CNN) to extract patch-level features, and finally adopts voting [18] or multiple instance learning (MIL) [19] to fuse different patches to establish a survival prediction model. A major limitation of the patch-based method is the lack of appropriate biological explanations of results when extracting patch features and merging patches. Some approaches seek to address this challenge by employing attention mechanisms [10,20]. On the other hand, there is more transparency and direct interpretability for nuclei-based approaches that aim to describe different cell populations and their spatial interactions. Such frameworks usually start with nuclei segmentation of WSI and then characterize nuclear morphology [21] or construct a multi-nuclear-type model to quantify intratumor heterogeneity [17]. With this approach, the tumor prognostic analysis is more dependent on contextual information and information about various cell types in tumor microenvironments. However, these nuclei-based models do not adequately consider the relationships between different cell types as well as within the same cell type, which means that these methods lack sufficient consideration of contextual information. Meanwhile, these models are mainly applied to predefined regions of interest rather than WSI owing to the need for greater computing resources.

Recently, several new studies on cancer prognosis using feature extraction have focused on segmented nuclei. For example, Lu et al. [22] extracted 615 features relating to nuclear shape, texture, and orientation disorder from nuclei obtained using watershed segmentation from each tissue microarray spot. Then, the 15 most prognostic quantitative histomorphometric features were identified and used to predict survival risk. In parallel, Alsubaie et al. [23] characterized the heterogeneity of LUAD by using morphological features of segmented tumor cell nuclei, then generated a heat map of the entire WSI and used Cox proportional risk regression models to find the most important discriminatory features to implement prognostic analyses. Another study by Chen et al. [7] was based on calculating partial eigenvalues for the cell nuclei to represent certain textural features, and ultimately selecting a subset of features for prognostic analysis. These models have some important limitations. First, while splitting the nucleus or extracting features from the segmented nucleus, the method of extracting features manually is not conducive to an automated pipeline. Second, there is no explicit classification of different types of nuclei, and a large amount of data is needed to effectively learn the characteristics of different cell types. Third, contextual information about the same or different types of nuclei is not fully utilized. Therefore, it is essential to develop a fully automatic pipeline to effectually utilize the contextual information of multiple cell types.

In this paper, we present a fully automated cellular-level dual-branch global fusion pipeline to predict survival based on WSI. Compared to nuclei-based prognosis methods, our pipeline can better utilize global contextual information, including different cell types and locations. Specifically, we first compute an embedded WSI map based on the segmentation and classification of all cell nuclei on the WSI, which maintains their spatial information. This approach has better interpretability compared to methods that compute patches or nucleus features extracted from some black-box mechanisms. Next, the features of the overall WSI and the features derived from relationships between different regions within the WSI are calculated separately for the WSI-embedded maps. The former features focus on global cell type and distribution, while the latter features focus more on contextual relationships between different or within the same cell types. Finally, these two types of global features are further fused to assess their implication for prognosis. We evaluated our pipeline using The Cancer Genome Atlas (TCGA) lung adenocarcinoma dataset and demonstrated promising performance. The proposed method is more robust compared to individual global features. Meanwhile, this overall framework has the potential to predict survival and treatment responses for other cancer malignancies.

## 2. Materials and Methods

### 2.1. Data Curation and Pre-Processing

**Figure 1:**
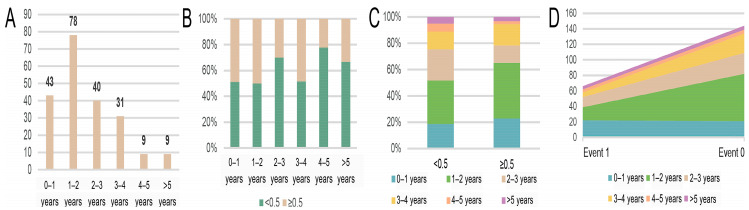
The distribution of dataset statistics. (**A**) represents the number of cases in different survival years; (**B**) shows the percentage of patients whose survival duration ended in the first half or the second half of the indicated year based on (**A**); (**C**) represents the number of cases per survival year based on (**B**); (**D**) shows the number of cases in each survival year for different survival events. Event is the observed state of survival, i.e., event 1 represents death and 0 represents a censoring event.

We applied the proposed methodology to the TCGA LUAD dataset, which was accessed from the TCGA data portal. Survival and phenotype information were obtained from UCSC Xena. The investigation focused on frozen section pathology images captured at a magnification of 20×, with corresponding overall survival (OS) data. Following data curation and rigorous quality control procedures, a cohort of 210 patients was meticulously assembled, with one WSI randomly selected from each patient. Figure 1 shows the OS distribution of the cases in the dataset, presented by year. Patients’ OS was concentrated between 0 and 4 years (Figure 1A). To randomly select training and testing cohorts, we then stratified patients according to whether their survival duration ended in the first half or the last half of a year. When stratified into half-year increments over the span of a year, the distribution of patients across these different OS intervals was relatively balanced (Figure 1B,C). Figure 1D underscores the equitable distribution of patients between the two survival events, where event 1 represents death and 0 represents a censoring event, except for those falling within the 1- to 2-year OS interval.

To establish a balanced partitioning, wherein the training and testing cohorts exhibited comparability across key characteristics such as gender, age, race/ethnicity, TNM categories, overall stage, and OS [24], the utilization of the propensity score-matching algorithm was instrumental in splitting the five-fold dataset. This rigorous approach ensured that our subsequent analyses were conducted on a well-matched and representative subset of the initial patient cohort. When the five-fold cross-validation was applied to divide the current dataset, the OS distributions for each fold were similar.

### 2.2. Overview of the Proposed Pipeline

**Figure 2:**
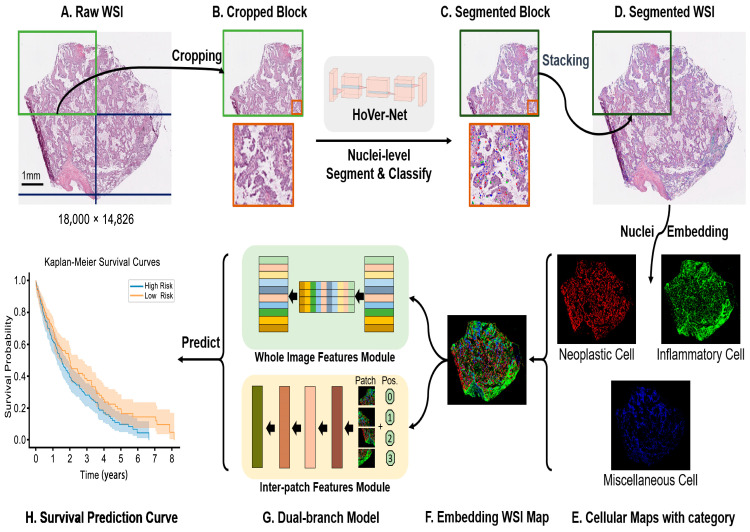
Overview of the proposed pipeline for survival prediction. (**A**,**B**): Cropping a whole-slide image (WSI) into multiple blocks. (**B**,**C**): Nuclei-based segmentation and classification. (**C**,**D**): Stacking segmented blocks as a segmented WSI. (**D**,**E**): Generating different types of nuclear maps. E-F: Embedding cell categories on the WSI map. (**F**,**G**): Extracting features based on the dual-branch model. (**G**,**H**): Prediction of survival risk from final features.

Figure 2 shows the proposed automatic segmentation of nuclei and the overall process of its application in survival prediction that mainly includes block-based nuclei segmentation and classification on WSI, aggregating the composition and structure of different cells, embedding those locations and class categories in the embedded WSI map, and extracting features from the embedded WSI map based on a dual-branch feature network to construct a survival prediction model. These steps are detailed in the following sections.

### 2.3. Block-Based Nuclei Segmentation

Transfer learning has shown outstanding performance in the field of deep learning [25,26]. We harnessed the power of transfer learning by employing the HoVer-Net architecture [27], which has been pre-trained on the PanNuke dataset [28] for both nuclei segmentation and cell type classification tasks. Multiple works have demonstrated the effectiveness of segmentation on the TCGA LUAD dataset directly using the pre-trained model without fine-tuning [29,30,31,32]. For example, Chen et al. [32] qualitatively assessed the results of segmentation by three pathologists, in which an accuracy of 0.902 was achieved for cell classification. Traditional methodologies for WSI analysis conventionally involve an initial step of identifying tissue regions, followed by the application of machine learning models to these designated areas. However, this conventional approach has several practical challenges. Many existing tissue-detection algorithms struggle to achieve robust generalization on WSI. Consequently, recourse to manual hyperparameter adjustments becomes necessary to attain satisfactory outcomes. To deal with this predicament, we introduced a novel block-based strategy tailored for the effective handling of gigapixel WSI. Meanwhile, the model-cropped WSIs into multiple blocks enables parallel computation to speed up inference due to computational efficiency and memory constraints. Moreover, we cropped the blocks without overlap in large sizes to minimize the effect of the border. Our approach entails the segmentation and labeling of cells within discrete sections of the WSI. To implement this, we partition each given WSI into smaller blocks (Figure 2B), with a predefined block size of 8000 × 8000 pixels. Subsequently, we employ the HoVer-Net on these individual blocks separately, enabling the precise segmentation and labeling of cellular entities (Figure 2C).

Upon attaining outputs from the block-wise processing, we stack the blocks to generate WSI maps with comprehensive cell classification (Figure 2D). Altogether, the categories of cells present on the lung adenocarcinoma slides encompass six distinct classes based on the PanNuke dataset, specifically neoplastic, non-neoplastic epithelial, inflammatory, connective, dead, and non-nuclei categories. In the context of our study, we excluded the non-nuclei category, retained the neoplastic and inflammatory categories, and merged the non-neoplastic epithelial, connective, and dead categories into a consolidated “miscellaneous” category. Subsequently, the segmented nuclei were allocated to three distinct categories, denoted as neoplastic (tumor), inflammatory (immune), and miscellaneous (Figure 2E). It should be noted that our results are calculated using direct inference from HoVer-Net and do not require additional annotations for re-training the model.

**Figure 3:**
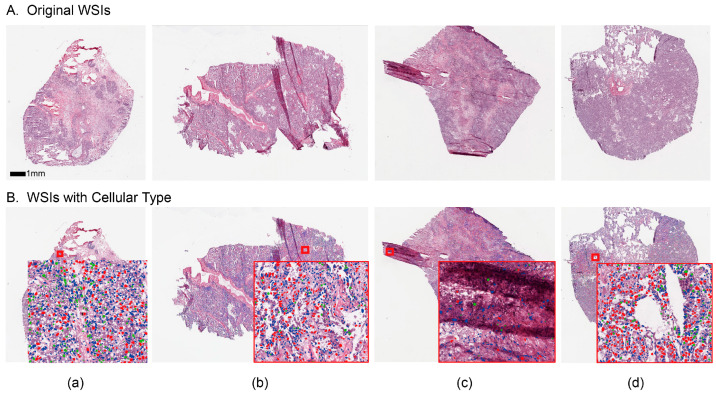
Examples of whole-slide images (WSI) without and with cellular type annotations, shown by magnification. (**A**) shows the original WSIs, and (**B**) shows the segmented WSIs with cellular type, in which (a), (b), (c) and (d) represent different samples.

Thereby, we can compute the WSI with the nuclear annotation from the raw WSI, as shown in Figure 3. Among them, columns (a) and (b) are sampled from the general position and zoomed in on the local detail map, which shows that the segmentation achieves excellent performance and most of the cells are annotated. The sample of magnified area in column (c) is taken from the folded position and shows that the annotations predominantly appear outside of this artifact region. Similarly, in column (d), the model avoids holes in the tissue. This shows that the algorithm has strong robustness to image variations. These outcomes lay the basis for subsequent accurate prognostic analysis.

### 2.4. Cellular Embedding of Segmented WSI

A WSI, scanned at 20× (0.50 µm/pixel), typically has billions of pixels, which usually contain over a million cells. Extracting cell features from this huge-scale WSI and visualizing them is still challenging. Since we are more focused on the global architecture and distribution of different cell populations than on the contours and textures of individual nuclei, our approach is to compress the local nuclear information to embed cell types in the WSI. First, we encode the abundances of the three cell types (neoplastic, inflammatory, and miscellaneous) for each 64 × 64 pixel window in three separate channels following Chen et al. [7]. Then, an embedded map of different cellular types (Figure 2F) with a 64-fold dimensional reduction from the raw WSI is obtained. We chose a down-sampling factor under 64 because cell images are commonly cropped to 256 × 256 pixels [33,34,35].

### 2.5. Dual Global Fusion for Survival Prediction

Figure 2G shows the process of feature extraction and prognostic analysis for embedded WSI maps. This model mainly consists of dual branches; one of the modules focuses on extracting global features from the maps, and the other focuses on extracting contextual features between inter-patches cropped from embedded maps. These two types of features are then combined to predict the patient prognosis.

Specifically, to compute the global features of an embedded WSI map, a network, $N_{whole}$, is built based on the DeepMixer [36]. This tool is a newly proposed simple network architecture based on multi-layer perceptron (*MLP*), which achieves competitive performance in image classification tasks. Since its architecture comprises mainly *MLP*, it is more conducive to learning global features [37,38]. The $N_{whole}$ handpicks only the feature extraction module from the MLP-Mixer, removing the classification projection headers according to our task. Each MLP block contains two fully connected layers and a nonlinearity Gaussian error linear unit [39] applied to each row independently of its input data tensor. One Mixer layer can be written as follows:

(1) $$M=FC\left(GELU\left(FC\left(x\right)\right)\right),$$

(2) $$MC=M\left(LN\left(X^{S\times C}\right)\right),$$

(3) $$MP=M\left(LN\left(X^{C\times S}\right)\right),\;$$

(4) $$Z_{l}^{1}=\left(Z_{l-1}^{1}+MP\left(Z_{l-1}^{1}\right)\right)+MC\left(Z_{l-1}^{1}+MP\left(Z_{l-1}^{1}\right)\right),\;\;\;\;l\in \left(1,\;\ldots ,\;L\right)$$

where x is the input, LN is layer normalization [40], L is the total number of Mixer blocks in $N_{whole}$, and M denotes an MLP block, which returns the output of the same dimension as the input by specifying the hidden layer dimension. MC denotes a channel-mixing *MLP*, which accepts input with a resolution of S×C, and similarly, MP is a patch-mixing *MLP* that accepts input images of C×S resolution. The MC allows communication between different channels, and the MP allows communication between different spatial locations. The feature reuse mechanism aims to reuse the upper layer $Z_{l}^{1}$. The final global feature is obtained after multiple Mixer layers in branch one.

The second branch applies ViT [41], a visual framework based on the self-attention mechanism with outstanding performance for handling contextual information. Similarly, we extract only the feature coding module as the branch $N_{inter}$, removing the classification headers. Specifically, the input X is first tokenized by reshaping as a sequence, which consists of all flattened 2D patches $x_{p}$, where p is the patch number. To define the spatial position of different patches and learn the contextual information from inter-patches, the position embeddings are added to the patch embedding as follows:

(5) $$Z_{0}^{2}=\left[x_{1}E;x_{2}E;\ldots ;x_{p}E\right]+E_{pos.},$$

where E is the linear projection and $E_{pos.}$ denotes the position embedding. Then, the initial embedding $Z_{0}^{2}$ is fed into the transformer layer to learn the contextual information from inter-patches. The transformer layer consists of multi-head self-attention (*MSA*) and *MLP*, which can be formulated as follows:

(6) $$Z_{l}^{2}=MSA\left(LN\left(Z_{l-1}^{2}\right)\right)+Z_{l-1}^{2}+MLP\left(MSA\left(LN\left(Z_{l-1}^{2}\right)\right)+Z_{l-1}^{2}\right),\;\;\;\;l\in \left(1,\;\ldots ,\;L\right)$$

where the calculation of *MSA* is as previously described [42]. After the L layer calculation, the final context feature with attention weight $Z_{l}^{2}$ is obtained in branch two. Finally, we concatenate two features with different focuses and add patient survival events as the final embedded WSI map features $Z=\left[Z_{l}^{1},{\;Z}_{l}^{2},\;\mu \right]$.

In a general survival analysis, patients are usually grouped into two categories: censored and non-censored. We use a binary μ∈(0, 1) to represent the survival events under observation. A censored case means the patient did not experience death during the follow-up period, which denotes they had survived longer than the recorded time. We predicted survival risk based on continuous survival data, differing from classification-based prognostic models. The proposed model directly generates survival risks r corresponding to patients i based on the embedded WSI map features. Hence, our loss function is defined as negative Cox log partial likelihood [43] based on the survival time t as follows:

(7) $$Loss\;\left(r_{i}\right)=\sum_{i}^{N}\mu_{i}\left(-r_{i}+log\sum_{j:t,\;j\geq t_{j}}\exp\left(r_{j}\right)\right).$$

## 3. Results

### 3.1. Experiment Implementation Details

For the final prediction of the extracted features, we added a fully connected layer in which the size of the input vector was 257. For each branch of the proposed method as well as the comparative methods, we used the same output length, i.e., 128. The depth of each branch was 2. The feature map as an input of the survival prediction model needs to be of a fixed size. We defined the input resolution as 256. Meanwhile, routine data augmentations were applied to our input data in our experiments, such as right-angle rotation, scaling, flipping, and transformations in brightness, contrast, and saturation. The patch size of the dual branch was set as 8. For the training, the optimizer was Adam, with a 1 × 10^−4^ learning rate, 5 × 10^−3^ weight decay, and 0.99 momentum. The batch size and epoch were 64 and 100. We initialized the weights with a fixed random number for initialization. We trained models until the last epoch as a stopping criterion. All models were implemented using PyTorch (version 1.12.1), and all training processes were trained on the two NVIDIA A100 SXM4 40GB graphics processing units in Linux (version 5.4.0-153-generic).

### 3.2. Evaluation of Prognosis Performance

**Figure 4:**
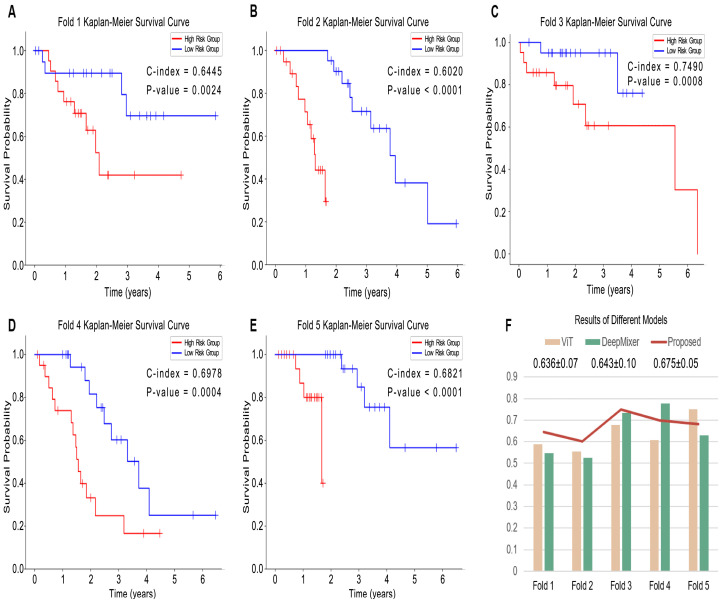
Survival analysis using the proposed methods and its constituent methods. (**A**–**E**) shows the results of our proposed method in every fold. (**F**) compares the C-indexes of different methods and includes the mean and standard deviation for each method.

We first evaluated the performance of the proposed pipeline. Based on the quantitative risk output from the prediction model, we divided patients into low-risk and high-risk groups using the optimal cut-off derived from the quantile classification scheme [44]. The cut-off values were calculated based on all samples in the training or validation datasets. The Kaplan–Meier estimator and two-sided log-rank test were used to evaluate the significance of patient stratification, and Antolini’s concordance index (C-index) was used to measure the goodness-of-fit of the model discriminative ability [45]. Figure 4A–E illustrates the Kaplan–Meier survival analysis results for each fold model. It can be seen that the *p*-values between our high-risk group and low-risk group are close to or less than 0.0001 in each fold, which was statistically significant. This shows not only that the data split is reasonable but also that the proposed method is robust.

Among the five folds, the third fold showed the highest C-index value (0.749), and the second and fifth folds had the lowest *p*-values (both below 0.0001). In the third fold, the results showed that both the high-risk and low-risk groups had a lower survival risk as well as survival times compared to the other folds. Of note, this indicates that our model is more effective in predicting consistent outcomes for patients who have longer survival times. This same finding was validated in the distribution of survival risk in the fourth and fifth folds. In the first fold, there was better differentiation between the low-risk and high-risk groups of results in some patients with longer survival times, while the stratification was not consistent for some patients with shorter survival times. Overall, the performance of all models showed significance in stratifying high-risk and low-risk groups.

Next, our proposed method comprised two constituent models, so we used two different models, ViT and DeepMixer, for our ablation and comparison experiments. As shown in Table 1, the average C-index of our proposed method (0.675) was higher than that of the others. Our method also achieved a higher C-index in the first through third folds. In the fourth fold, our method’s C-index was lower than DeepMixer’s but higher than ViT’s; in the fifth fold, it was lower than ViT’s but higher than DeepMixer’s, as shown in Figure 4F. The proposed method’s C-index also had a lower standard deviation. The results show that our dual-branch structure has better robustness and can compensate for the performance deficiencies of the two branches separately. Furthermore, this gives us an enlightening direction to improve our work in the future. We anticipate making full use of the advantages of the different branches to improve the final performance.

### 3.3. Feature Visualization Analysis

To further evaluate the performance of the proposed method, we performed a visual study based on the characteristics of each patient. The following analysis unifies the visualization technique using the Uniform Manifold Approximation and Projection (UMAP) [46] tool to understand the underlying data structure. The neighbor number is the most critical parameter of UMAP, which effectively controls how UMAP balances local versus global structure. Low numbers of neighbors will push UMAP to focus more on the local structure by constraining the number of neighboring points considered when analyzing the data in high dimensions. Hence, we conducted two group experiments where the neighbor numbers were equal to 4 and 5 for each visualization. Each column represents the result of a different fold. The corresponding label of the color bar is the survival years.

**Figure 5:**
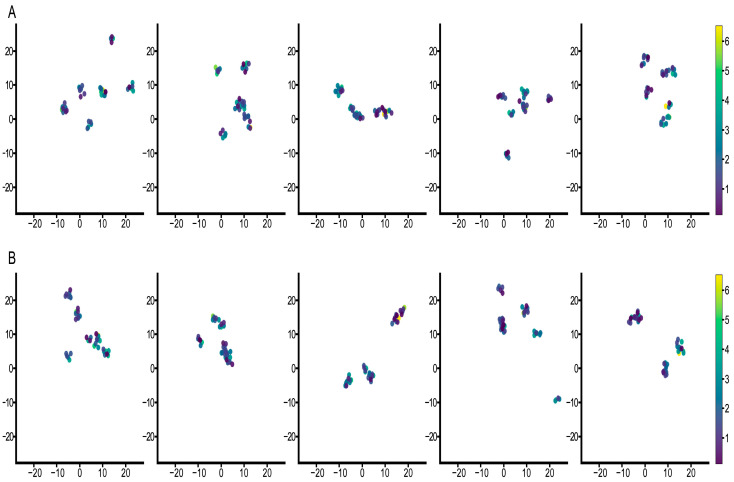
UMAP visualization for analysis of survival probability. (**A**) shows the results with the number of neighbors equal to 4, and (**B**) shows results with the number of neighbors equal to 5. Each column represents the result of each fold. Colors indicate survival time.

First, we analyzed the survival risk as shown in Figure 5. We observed that both groups had good separation where no complete overlap was noticed between the samples. Samples with similar survival years were all in the same cluster, and each cluster kept a distance from other clusters. This distinct clustering is crucial for model performance for predicting survival probabilities. When the neighbor number was 4, the number of samples in each cluster was closer to the survival years and the boundaries between different clusters were sharper compared to when the neighbor number was 5, but the latter had a more substantial clustering effect. These results indicate that the proposed method predicts survival probability relatively accurately.

**Figure 6:**
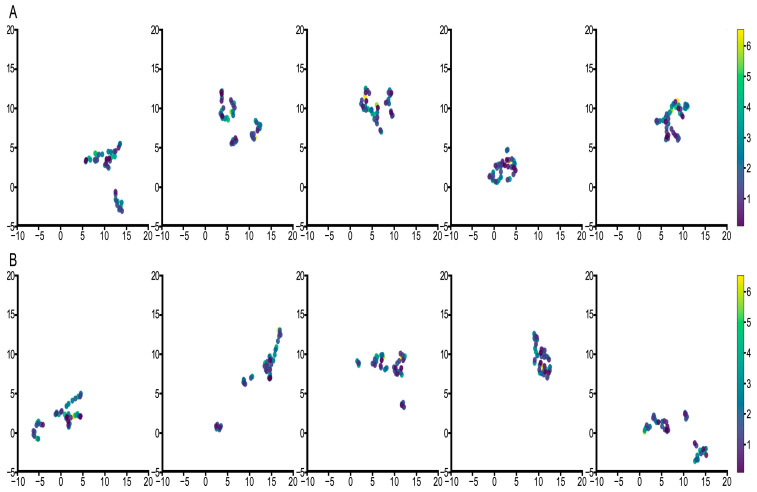
UMAP visualization for analysis of embedded WSI map features when the neighbors’ number is 4. (**A**) represents the result for the feature without survival event, and (**B**) represents the feature with survival event. Each column represents the result of each fold.

**Figure 7:**
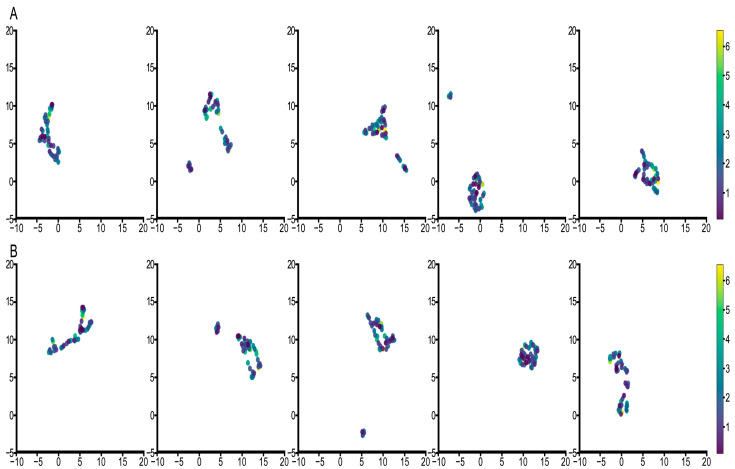
UMAP visualization for analysis of embedded WSI map features when the neighbors’ number is 5. (**A**) represents the result for the feature without survival event, and (**B**) represents the feature with survival event. Each column represents the result of each fold.

Next, we analyzed the final embedded WSI map features Z and those without survival events. Figure 6 and Figure 7 show the results of the two groups’ visualizations, respectively. The results for both groups show that features with events had better differentiation and more pronounced clustering. A linear relationship between samples in the features with the events was perceived which we hypothesized to be particularly beneficial for optimization based on the loss function of survival ranking. This linearity also demonstrates that survival events are deeply integrated into the model and are instrumental in predicting the final probability of survival. Additionally, we also found that better clustering results were obtained and that the cluster number was closer to the survival years when the neighbor number was 4, which was consistent with the previous analysis of survival probability visualization. These results show that the features learned by the model are effective and are better predictors of patient survival probability compared to other models.

## 4. Discussion

Here, we present a fully automated cellular-level dual-branch survival prediction pipeline for lung adenocarcinoma pathologic images, which achieved promising results using the LUAD dataset of TGCA. Compared to black-box methods that directly use pathologic images, our proposed method uses more interpretable images at the nucleus level for survival analysis, which makes the results more convincing and comprehensible. Some kernel-based methods partly use manual feature extraction, which means that full automation has not been achieved. These methods extract features directly with convolution, which is more concerned with local regions and exhibits general limitations on modeling explicit long-range relations [47]. These methods not only are unfavorable for learning global features but also have difficulty learning features between cells that are far away from each other.

To address these challenges, we first segment and classify the nuclei from raw WSI. After performing category screening, we generate an embedded WSI map from the segmented images of cell nuclei. The embedded map more directly demonstrates the distribution of various types of cells in the tissue, especially neoplastic cells and inflammatory cells, which are indispensable for the analysis of the tumor microenvironment. The three categories of cells are aggregated into the final embedded WSI map. We also perform 64× down-sampling to make subsequent survival prediction more efficient. The generated embedded maps are then fed into our dual-branch network. One branch learns global features and the other learns inter-patch features, which only consider the global distribution of various types of cells, but also allow for the computation of long-distance cellular correlations. Subsequently, we fuse these two features from different branches for the final survival prediction, which enables complete automation of survival analysis.

To demonstrate the proposed method, we performed several experiments using the LUAD dataset. First, the model was cross-validated with five folds, and survival Kaplan–Meier curves were plotted separately for each predicted outcome in two groups, high and low risk, and the C-index was calculated. The models all achieved good performance, and the results of the analysis revealed that our results in each fold were statistically significant. Ablation and comparison studies with other methods showed that the proposed method had a higher average consistency, and less variance in the results of different folds, demonstrating the robustness of the proposed method. Furthermore, visualization of the survival probabilities and the final embedded WSI map features revealed a clustered relationship between the probability predicted by our method and the survival years, as well as a great predictive performance of the embedded map features extracted using our method. Our method provides a fully automated dual-branch-based pipeline for survival prediction using cellular-level information only, which will be valuable in clinical applications.

Our study has several limitations. First, the computational cost of WSI nuclei segmentation and classification is relatively high, and further investigation on how to quickly infer the segmentation results at larger batch sizes is needed. Second, the current prognostic model is validated on a smaller-scale dataset; future validation is planned with other, larger LUAD datasets. Third, this study focuses on predicting prognosis in patients with pathologic images; how to apply our approach to multimodal data such as genes for survival prediction is well worth exploring. Finally, in the proposed dual-branch network, we use a concatenate strategy to fuse different features, and how to merge such features dynamically and adaptively is an important future research direction.

## 5. Conclusions

This paper presents a fully automated dual-branch-based cellular-level survival prediction pipeline for lung adenocarcinoma pathologic images. This method generates embedded WSI maps for intelligent automated survival analysis by flexibly describing the composition and structure of different cell populations on WSI. This allows us to investigate different cell populations as well as the tumor microenvironment within gigapixel WSI. Furthermore, we use a dual-branch model to learn whole-slide and inter-patch features of cell distribution, allowing the network to sufficiently learn different types and different locations of cells. We demonstrate the effectiveness and robustness of the proposed method with superior experimental results on the LUAD dataset compared to other models. The proposed method is a general WSI-based learning pipeline, which allows researchers to explore the clinical prognosis for predicting different cancer types. In the future, we will expand techniques based on the proposed model to integrate embedded maps with genetic data and improve survival prediction.

## Figures and Tables

**Table 1:** Performance comparison with other methods.

Methods	C-Index	*p*-Value
ViT	0.636 ± 0.07	1.8 × 10^−6^
DeepMixer	0.643 ± 0.10	3.7 × 10^−5^
**Our**	**0.675** ** ± 0.05**	**5.6 × 10^−5^**

## Data Availability

All the data are available from the public databases, including the TCGA database (https://portal.gdc.cancer.gov/, last accessed on 30 January 2022) and UCSC Xena (https://tcga.xenahubs.net/, last accessed on 30 January 2022).
